# Antimicrobial and Antibiofilm Activity of Auranofin and Its Two Derivatives Bearing Naproxen and Acetylcysteine as Ligands Against Staphylococci †

**DOI:** 10.3390/antibiotics14020118

**Published:** 2025-01-23

**Authors:** Caterina Ferretti, Lorenzo Chiaverini, Noemi Poma, Andrea Dalli, Riccardo Di Leo, Laura Rindi, Alessandro Marrone, Iogann Tolbatov, Diego La Mendola, Arianna Tavanti, Tiziano Marzo, Mariagrazia Di Luca

**Affiliations:** 1 Department of Biology, University of Pisa, 56126 Pisa, Italy; c.ferretti15@studenti.unipi.it (C.F.); noemi.pomasajama@unipi.it (N.P.); a.dalli@studenti.unipi.it (A.D.); arianna.tavanti@unipi.it (A.T.); 2 Department of Translational Research and New Technologies in Medicine and Surgery, University of Pisa, 56126 Pisa, Italy; laura.rindi@unipi.it; 3 Department of Pharmacy, University of Pisa, 56126 Pisa, Italy; lorenzo.chiaverini@phd.unipi.it (L.C.); riccardo.dileo@phd.unipi.it (R.D.L.); diego.lamendola@unipi.it (D.L.M.); 4 CNR IFC, Institute of Clinical Physiology, National Research Council of Italy CNR Research Area, 56124 Pisa, Italy; 5 Department of Pharmacy, University of Chieti-Pescara “G. D’Annunzio”, 66100 Chieti, Italy; amarrone@unich.it; 6 Department of Chemical, Physical, Mathematical and Natural Sciences, University of Sassari, 07100 Sassari, Italy; tolbatov.i@gmail.com

**Keywords:** AMR spread, auranofin, auranofin analogues, naproxen, acetylcysteine, *Staphylococcus aureus*, *Staphylococcus epidermidis*, biofilm

## Abstract

**Background/Objectives**: The ability of bacteria to form biofilms makes them more tolerant to traditional antibiotics. Given the lack of new antibiotic development, drug repurposing offers a strategy for discovering new treatments. Auranofin (AF), a gold-based compound indicated for the treatment of rheumatoid arthritis, shows promising antibacterial activity. This study investigates the antimicrobial and antibiofilm activity of AF and its two derivatives in which the thiosugar ligand is replaced by acetylcysteine (AF-AcCys) or naproxen (AF-Napx), against *Staphylococcus aureus* and *Staphylococcus epidermidis*. **Methods**: AF was conjugated by transmetalation with either naproxen or acetylcysteine. Assessments of their stability in DMSO/H_2_O and lipophilicity expressed as the LogP were performed. The antimicrobial activity of AF and its analogues were investigated by broth microdilution assay to determine the minimum inhibitory concentration (MIC) and versus biofilm to obtain the minimum bactericidal biofilm concentration (MBBC) and minimum biofilm eradication concentration (MBEC). **Results**: AF derivatives were found to be stable in a DMSO/H_2_O mixture for 48 h. AF-Napx showed a LogP = 1.25 ± 0.22, close to AF, while AF-AcCys had a LogP = −0.95. MIC values of *S. aureus* and *S. epidermidis* were ranging from 2 µM to 0.25 µM, and ≤0.12 µM, respectively. Both AF and AF-Napx maintained efficacy against biofilm-embedded *S. aureus* and *S. epidermidis* at non-cytotoxic concentrations, with AF-Napx demonstrating lower MBBC values for *S. epidermidis*. **Conclusions**: AF, and especially its naproxen conjugate, holds potential as a therapeutic agent for treating biofilm-associated infections caused by *S. aureus* and *S. epidermidis*, particularly in device-related infections where both infection and inflammation are present.

## 1. Introduction

Antimicrobial resistance (AMR) is associated with a high risk of treatment failure and the relapsing of infections. Due to its increase in incidence, AMR has become a highly relevant health, clinical, and economic issue, being indeed considered one of the greatest challenges in medical practice nowadays [1]. The antibiotic resistance crisis has been attributed primarily to the overuse and misuse of antibacterials which determined the selection of non-susceptible bacterial clones. In addition, the lack of new antimicrobial drugs’ development by the pharmaceutical industry due to reduced economic incentives will lead to a period where antibacterial choices could be extremely limited [2]. The increasing presence of drug-resistant bacteria is also caused by the ability of pathogens to form biofilms. In particular, staphylococcal biofilm is involved in around 80% of chronic infections, and it plays a principal role in 60% of all human bacterial infections [3]. Biofilm-associated bacteria growing in sessile form are able to tolerate up to 1000 times higher concentrations of antimicrobial agents compared to their planktonic counterparts, making biofilm-related infections extremely difficult to treat [4]. In this scenario, new antibiofilm strategies are necessary.

Gold-based complexes, especially gold (I) and gold (III), are emerging as a class of compounds capable of effectively dealing with bacterial infections. In particular, auranofin and a number of its derivatives, bearing different ligands in place of thiosugars, showed really promising antimicrobial activity against several strains of Gram-positive bacteria [5,6,7]. Auranofin (AF) ([1-(thio-κS)-β-d-glucopyranose-2,3,4,6-tetraacetato](triethylphosphine)-gold) is a gold(I)-containing linear complex bearing triethylphosphine and thioglucose tetraacetate as ligands. It was approved in 1985 by the Food and Drug Administration (FDA) as a first-line treatment against rheumatoid arthritis, but to this date its clinical use has been mostly replaced by synthetic and biological disease-modifying antirheumatic drugs (DMARDs), such as methotrexate, hydroxychloroquine, sulfasalazine, and adalimumab, due to their generally greater efficacy [8,9]. Despite this, AF has been widely repurposed for use in anticancer, antibacterial, antiviral, and antiparasitic treatments, and it is also involved in several clinical trials. Its potential as an antibacterial agent has proven to be especially promising. AF demonstrates high efficacy against Gram-positive bacteria, including those resistant to multiple drugs, like *Staphylococcus aureus*, *Enterococcus faecium*, and *Enterococcus faecalis*, while showing low activity against Gram-negative strains [7]. The pharmacological effect of AF is attributed to the [AuPEt_3_]^+^ cation, considered the true pharmacophore, which forms following the release of the thiosugar moiety in the biological environment. The high affinity of gold(I) for thiols and selenols makes this drug and its derivatives capable of strongly interacting with proteins containing such groups, thus impairing key pathways fundamental for cell survival. In fact, it has been demonstrated how AF and its analogues can inhibit the growth of parasites, bacteria, and cancer cells by targeting the thioredoxin reductase (TrxR), causing an accumulation of ROS inside the cell and eventually inducing its death [10,11]. Experiments, based on high-resolution mass spectrometry, highlighted how the [AuPEt_3_]^+^ moiety can bind to the cysteine and selenocysteine groups present in a synthetic peptide mimicking the redox-active site of thioredoxin reductase (dTrxR(488–499)) [6,12]. Moreover, Jackson-Rosario and colleagues [13] showed how AF can react and form a stable complex with hydroselenyl radicals (HSe^−^) that are crucial species for the selenoprotein synthesis. The formation of such a complex can probably lead to the reduction in the selenium pool available for the cells, thereby impairing the synthesis of selenium-containing proteins and, in turn, the growth of the organisms requiring them for their metabolism [13].

Given the ascertained biological activity of AF, its conjugation with different molecules may improve its antibacterial action, providing new pharmacological properties with improved efficacy and/or synergistic effects. For example, it has been demonstrated that AF conjugation with *N*-acetylcysteine, a molecule with anti-inflammatory properties, exhibited antibiofilm activity [14,15,16]. *N*-acetylcysteine is useful for the treatment of chronic infections, characterized by a high level of local inflammation [17]. In addition, it has been included in the World Health Association (WHO) List of Essential Medicines and is used in a variety of treatments against disorders like cystic fibrosis, chronic obstructive pulmonary disease, chronic bronchitis, and several others [18]. Another alternative molecule that can be used for the AF conjugation is naproxen, which is an anti-inflammatory drug belonging to the Non-Steroidal Anti-Inflammatory Drugs (NSAIDs) group, showing analgesic and antipyretic properties [19]. Since the conjugation of a bioactive molecule to a metal fragment like [AuPEt_3_]^+^ has proven a valid strategy for the development of potential new drugs [6,20], here, we investigated the antimicrobial activity of AF alone and two of its analogues conjugated with either naproxen (AF-Napx) or acetylcysteine (AF-AcCys) versus in vitro biofilms of *S. aureus* and *S. epidermidis* clinical isolates.

## 2. Results

### 2.1. Synthesis, Characterization, In-Solution Stability and LogP Evaluation of AF-Napx and AF-AcCys

**Figure 1:**
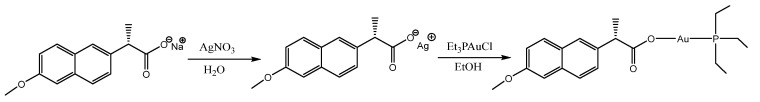
Reaction scheme for the preparation of AF-Napx.

The novel gold(I) carboxylate complex bearing the [AuPEt_3_]^+^ moiety bound to naproxen was synthesized and characterized by ^1^H, ^31^P, ^13^C NMR spectroscopy (Figures S1–S3), mass spectrometry (HR-ESI-MS), and elemental analysis. The compound was obtained by transmetalation, reacting Et_3_PAuCl with the silver salt of naproxen, as depicted in Figure 1. This latter compound was obtained by reacting the corresponding sodium salt with AgNO_3_ in the dark. This synthetic strategy has already been used successfully for the obtainment of similar gold (I) carboxylate complexes [21,22]. In particular, Johnson et al. recently described a series of gold(I) carboxylate complexes with triphenylphosphine and various NSAIDs as ligands, synthesized using this procedure, and investigated for their anti-breast cancer stem cell properties [23]. The reported yields for these compounds were approximately 65% (64% for the complex with naproxen as ligand) which are comparable to the 55% yield achieved by us for AF-Napx.

The coordination of naproxen to the [AuPEt_3_]^+^ moiety was confirmed by the relative high-field shift in the ^31^P-NMR signal corresponding to the coordinated triethylphosphine, in comparison with the precursor (25 ppm compared to 32 ppm of Et_3_PAuCl in CDCl_3_, Figure S3). The presence of a ^31^P-NMR signal in this region (27–25 ppm) is diagnostic for these kind of complexes [23,24]. In the ^13^C and ^1^H-NMR spectra, all the expected signals for the complex are present. In the ^1^H-NMR spectrum, multiplets are observed at 1.80 ppm and 1.17 ppm, corresponding to the CH_2_ and CH_3_ groups of the triethylphosphine ligand coordinated to gold(I), respectively. In the ^13^C{^1^H}-NMR spectrum, the peaks accounting for the same groups are present at 18 ppm and 9 ppm, respectively, while the carboxylate group is observed at 180 ppm. The HR-ESI-MS analysis further verified the obtainment of the desired product (Figure S6), and the elemental analysis results are also consistent with the expected compound, also confirming its purity.

Similarly, the AF-AcCys derivative, previously synthesized by us [6] following an already reported procedure that involves reacting acetylcysteine with Et_3_PAuCl in the presence of NaOH [25], was also characterized by multinuclear NMR spectroscopy (^1^H, ^31^P, ^13^C), mass spectrometry (HR-ESI-MS, Figure S7), and elemental analysis, providing further confirmation of its structure and purity.

The stability of AF-Napx was evaluated by both UV–Vis spectroscopy and ^31^P{^1^H}-NMR experiments in a mixture of DMSO/H_2_O (6:4). Firstly, the UV–Vis spectral profile of the AF-Napx complex showed no significant changes, confirming its stability in a mixture of organic solvent and water (Figure S4). Notably, the degradation percentage was minimal, with a value of 3.4% after 48 h. Similarly, the stability in the same mixture (DMSO/H_2_O) was spectrophotometrically assessed for AF-AcCys, also demonstrated stability over the same 48 h timeframe (Figure S5) with a degradation percentage of 2.1%. Moreover, the ^31^P{^1^H}-NMR experiments conducted on AF-Napx did not indicate the presence of any distinctive changes throughout the incubation period. However, as previously reported for AF-AcCys [6], an additional peak was detected along with the signal of the phosphane ligand in the neutral complexes, appearing at 47 ppm and corresponding to [Au(PEt_3_)_2_]^+^. The appearance of the new peak attributable to [Au(PEt_3_)_2_]^+^ is not surprising and consistent with a previous observation made on AF derivatives [26]. This species is formed owing to a well-known ligand scrambling reaction. Concerning its impact on the overall pharmacological activity, we can assume that, in the biological environment, its impact is negligible. Indeed, in these conditions, several biological binding partners are available to quickly react with the prodrug, hampering the formation of the [Au(PEt_3_)_2_]^+^ species that is operative only in cell-free NMR experimental conditions. In conclusion, the stability profile of these two complexes is very similar to each other, and these results are comparable to those of the parent drug AF, which is also stable under similar experimental conditions [26].

The lipophilicity of AF-Napx was assessed by measuring the octanol–water partition coefficient expressed as LogP. Lipophilicity is an important physicochemical parameter since it is related to membrane permeability, drug absorption, and distribution [6]. The LogP value measured for AF-Napx was 1.25 ± 0.22 indicating a lipophilic character of the complex. This value positions AF-Napx between AF-AcCys (LogP = −0.95) and AF (LogP = 1.60) on the lipophilicity scale, but with a closer similarity to the parent drug.

### 2.2. XTT Reduction Assay upon Cell Exposure to AF, AF-Napx, and AF-AcCys

**Figure 2:**
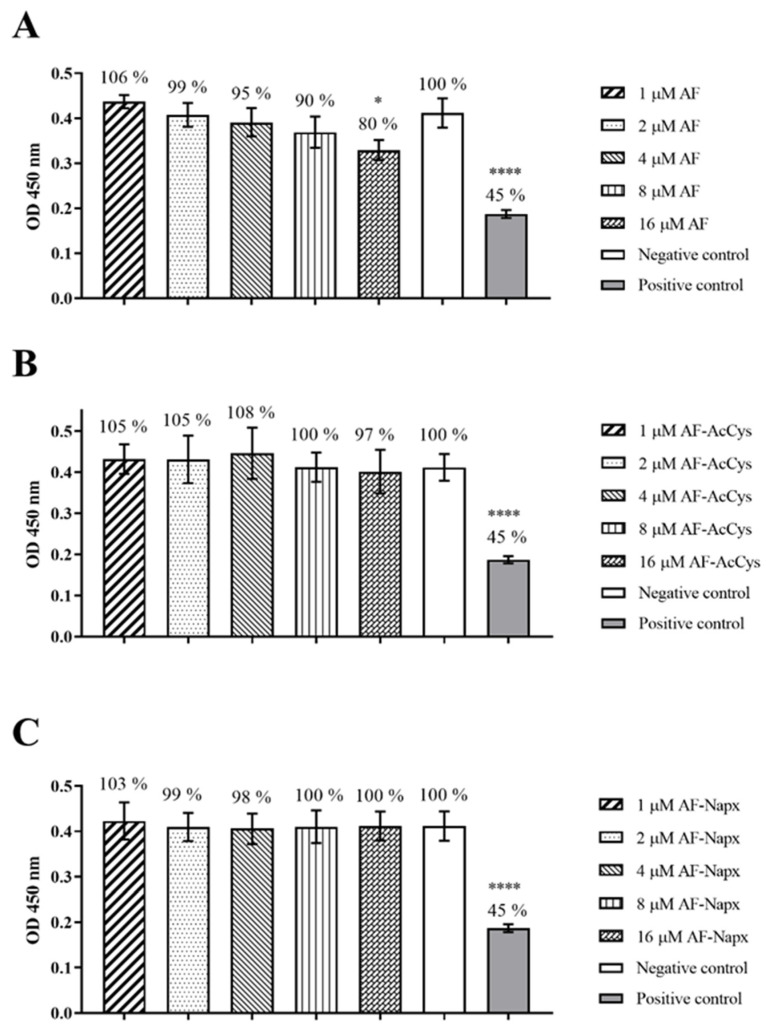
Absorbance values at 450 nm of formazan salt production by A459 cells exposed to different concentrations 1, 2, 4, 8, and 16 µM of AF (**A**), AF-AcCys (**B**), and AF-Napx (**C**). Culture media alone and 1% *v/v* Triton X-100 were included as negative and positive controls, respectively. Data are expressed as means ± SD of three independent experiments with three replicates each. * *p* ≤ 0.05, **** *p* ≤ 0.0001.

A549 cells were exposed for 24 h to different concentrations of AF, AF-AcCys, and AF-Napx (1, 2, 4, 8 and 16 µM), following this incubation period the metabolic activity was assessed by the XTT assay. Cells exposed to AF (Figure 2A) showed a slight reduction in cell viability at increasing concentrations of AF. However, no significant difference was observed up to 8 µM when compared to the negative control. Instead, cells, exposed to 16 µM of AF, showed a significant reduction in viability (80%) (*p* ≤ 0.05). Cell exposure to AF-Napx and AcCys (Figure 2B and Figure 2C, respectively) did not show any cytotoxic effect, as shown by the percentage of viability. Importantly, no significant difference was observed when compared to the negative control.

### 2.3. Antimicrobial Activity of AF and Its Derivates Against Planktonic and Biofilm Staphylococcal Strains

Antimicrobial susceptibility of the staphylococcal species used in this study, in both planktonic and in sessile forms, was tested for AF, AF-AcCys, and AF-Napx. The broth microdilution assay was performed following CLSI guidelines, as recommended for antibiotic compounds. As shown in Table 1, MIC values of AF ranged from 2 µM to 0.25 µM when tested versus *S. aureus* strains, while the MIC values observed for *S. epidermidis* were ≤0.12 µM.

The conjugation of acetylcysteine to AF determined MIC values similar to AF alone for *S. aureus* isolates, while higher values (2 or >2 µM) were obtained for *S. epidermidis* strains. Finally, AF-Napx exhibited MIC values ranging from 2 to 0.25 µM for all tested strains. Interestingly, the MIC values observed for AF (the most lipophilic compoundtested) were lower than the ones for AF-AcCys and AF-Napx in most cases.

The activity of AF, AF-AcCys, and AF-Napx was assessed against the staphylococcal strains growing as biofilms. Twenty-four-hour-old biofilm beads were treated with each antimicrobial agent in a range of concentration from 8 to 0.5 µM, then the bacterial concentration expressed as CFU/mL was calculated to determine the MBBC and MBEC for each tested isolate. MBBC and MBEC values of AF and its derivatives are shown in Table 2. MBBCs and MBECs of AF ranged from 8 to 0.5 µM for *S. aureus* strains, except for *S. aureus* 12, whose MBEC was >8 µM. For *S. epidermidis* clinical isolates, MBBC and MBEC values ranged from 8 to 2 µM. In the case of AF-AcCys, MBBC and MBEC values were >8 or 8 µM for all staphylococcal isolates, apart from the MBBC of *S. aureus* 12 that was 4 µM. AF-Napx MBBC values were 8 µM for *S. aureus* isolates, apart from *S. aureus* 11 (>8 µM), while MBECs were >8 µM for all the *S. aureus* tested strains. In the case of *S. epidermidis* isolates, AF-Napx MBBC, and MBEC values were lower (ranged from 2 to 1 µM) compared to *S. aureus* strains.

### 2.4. Theoretical Calculations

**Figure 3:**
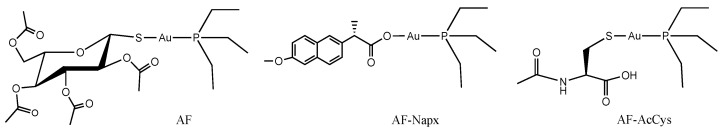
Structures of auranofin (AF) and its derivatives with naproxen (AF-Napx) and acetylcysteine (AF-AcCys).

Theoretical approaches were exploited to delineate the structural features responsible for the antibacterial response of the tiled Au(I) complexes (Figure 3). The experimentally detected trend of potency, AF-Napx > AF > AF-AcCys, at least when the activity against *S. epidermidis* based on MBBC and MBEC is concerned, is ascribed to either the release of the pharmacophore fragment [AuPEt_3_]^+^ or its binding at specific molecular targets. Indeed, among various mechanisms proposed for the antibacterial action of AF, the inhibition of the TrxR has been identified with [27], presumably via the coordination of the active fragment [AuPEt_3_]^+^ on to the catalytic selenocysteine residue of this enzyme. By assuming that the complex with the most facile release of this moiety is the most active, we used DFT approaches to investigate the fragmentation of the three auranofin derivatives consisting of the active moiety [AuPEt_3_]^+^ and the carrier moiety, either Napx, thiosugar, or AcCys. The calculated values of fragmentation energy (FE), bond dissociation energy (BDE), and bond dissociation free energy (BDFE) were reported in Table 3. All estimates evidenced the same trend, demonstrating that the release of the cationic complex [AuPEt_3_]^+^ from AF-Napx has the lowest energy cost, while the highest energy cost was ascertained in the case of AF-AcCys.

### 2.5. SEM Examination of the Antibiofilm Effect Produced by AF and Its Derivatives

**Figure 4:**
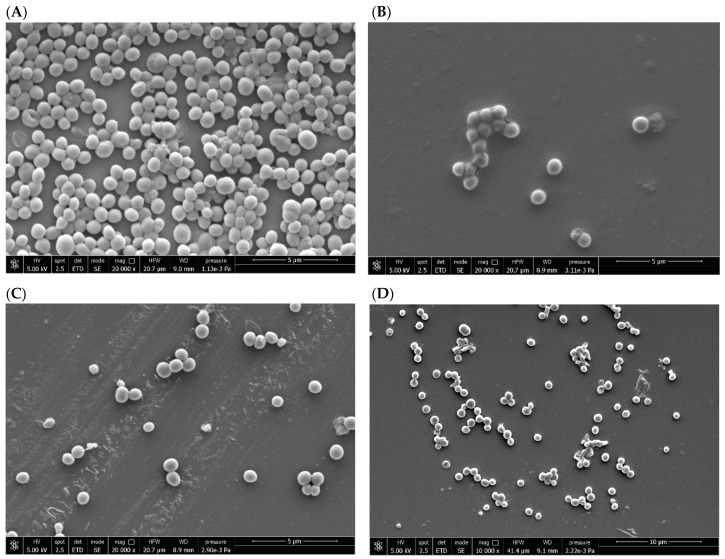
SEM imaging *S. aureus* ATCC43300 biofilm. Micrographs of sample slides, untreated (**A**) and treated with AF (**B**), AF-AcCys (**C**), and AF-Napx (**D**), can be observed. When *S. aureus* is treated with AF and AF-Napx, cellular damage occurs, resulting in morphological changes and complete lysis. The treatment with AF-AcCys causes the formation of a slim film that coats bacterial cells.

**Figure 5:**
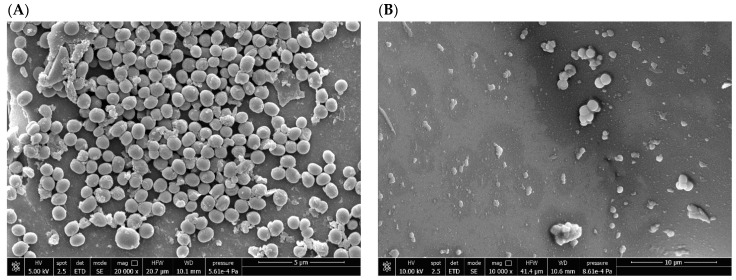

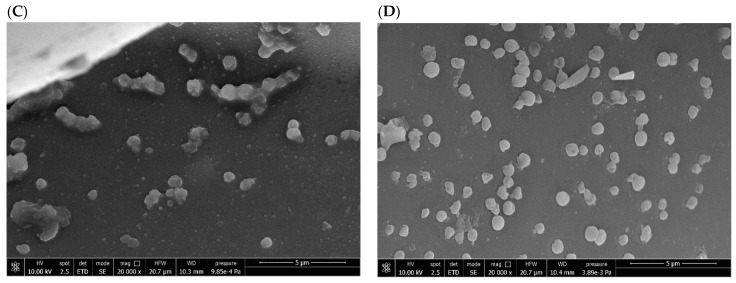
SEM imaging of *S. epidermidis* 7 biofilm. Micrographs of sample beads, untreated (**A**) and treated with AF (**B**), AF-AcCys (**C**), and AF-Napx (**D**), can be observed. When *S. epidermidis* is exposed to AF and AF-Napx, cellular damage ensues, causing morphological changes and complete lysis. The treatment with AF-AcCys leads to the formation of a thin film that covers bacterial cells.

SEM was used to analyze the effect of gold-based compounds at sub-MBBCs against *S. aureus* and *S. epidermidis* in sessile form for 24 h (Figure 4 and Figure 5). When compared to the untreated biofilm used as growth control, clear ultrastructural differences were observed in the bacterial cells treated with AF and its derivatives. Biofilm samples, used as untreated control (Figure 4A and Figure 5A), were characterized by coccus bacterial cells, adhering to each other and to the glass substrate. The exposure of bacteria to AF (Figure 4B and Figure 5B) led to a cellular damage, resulting in a morphological alteration and in a complete lysis for both bacterial species. A similar condition was observed when *S. aureus* and *S. epidermidis* biofilms were treated with AF-Napx (Figure 4D and Figure 5D, respectively), although the bactericidal effect was more evident in *S. epidermidis*, where the morphology of the cells suggested bacterial killing. Interestingly, as result of biofilm treatment with AF-AcCys, a slim film covering bacterial cells was formed in both *S. aureus* and *S. epidermidis* (Figure 4C and Figure 5C).

## 3. Discussion

Antibiotic resistance is considered by the WHO as one of the top ten threats to global health [28]. In 2021, 4.71 million deaths were predicted to be associated with bacterial AMR [29]. Among the leading pathogens whose attributable deaths are associated with antimicrobial resistance, methicillin-resistant *S. aureus* is included in the priority list by the WHO for which new antimicrobial solutions are needed [30].

Given that only a few pharmaceutical companies are investing in research and the development of new antibiotic compounds, drug repurposing accelerates the development of antimicrobial therapy regimes by leveraging pre-existing safety and efficacy data. In this context, AF, which is a metal-based gold drug and a TrxR inhibitor, has demonstrated potent bactericidal activity against Gram-positive bacteria, such as *S. aureus*, *E. faecium*, and *E. faecalis*, including strains that are resistant to conventional antimicrobials such as methicillin and vancomycin [7,31]. AF is known to damage the antioxidative defence and renders organisms more sensitive to oxidative stress; therefore, it has been proposed as a promising antimicrobial molecule.

As expected, AF was active against staphylococci at no cytotoxic concentrations, as previously described [6]. In particular, MIC values of AF against *S. aureus* ranged from 2 to 0.25 µM, corroborating previous published studies [32,33]. The drug was active at the lower tested concentration versus *S. epidermidis* (MIC values ≤ 0.12 µM), this behaviour could be explained based on the chemical–physical parameters of the tested molecules, including the LogP values. Indeed, AF is the most lipophilic compound of the tested panel. This property may in turn allow for higher activity toward *S. epidermidis* that has a more hydrophobic surface due to the chemical structure of the microbial cell wall [6].

Here, it was observed that AF conjugated with either acetylcysteine or naproxen was shown to be less cytotoxic compared to AF alone, still maintaining its antimicrobial activity at least against *S. aureus*. In contrast, the addition of either naproxen or acetylcysteine reduced the efficacy of AF versus *S. epidermidis*, with MIC values increasing by 5 to 10 times. However, it should be noted that these concentrations were still below the cytotoxic limit. In fact, AF is now approved for the long-term management of rheumatoid arthritis, with patients using the drug daily at a dose of 6 mg/day for more than 5 years, which is significantly longer than the standard antibiotic regimen of 1 to 2 weeks. Consequently, any toxicity linked to AF should not be a major obstacle to its repurposing as a new antibacterial treatment for staphylococcal infections [33].

Experimental evidence of the reduced toxicity of AF analogues in comparison with the parental complex was previously described; it was observed that sugar-modified analogues of AF [34] exhibited ameliorated inhibitory action against *H. pylori* and caused reduced toxicity to mammalian cells compared to AF, with IC50 values 4–6 times higher than AF. Similarly, AF analogues, synthesized by replacing the thiosugar structure with an aromatic or aliphatic thiol ligand, showed robust antibacterial activity against *S. aureus* and *E. faecium* up to seven times higher than that of AF simultaneously. Notably, these analogues exhibited similar or lower mammalian cytotoxicity compared with AF [35].

The anti-staphylococcal activity of AF was still maintained versus biofilm-embedded bacteria, both in *S. aureus* and *S. epidermidis* at non-cytotoxic concentrations. The antibiofilm activity of AF against the two species of *Staphylococcus* was also observed by Thangamani and colleagues [33], who treated *S. aureus* and *S. epidermidis* biofilm with 1 and 4 µg/mL AF, respectively, and observed a biomass reduction of 60–70%. In most cases, the biofilm bactericidal concentration was the same as the eradicating concentration. Usually, bacteria in the biofilm form are susceptible to very high concentrations of antibiotics or long therapy courses, which could be toxic for humans [36].

While AF conjugate with acetylcysteine did not show activity against biofilms of both staphylococcal species, AF-Napx was active against biofilm-embedded *S. epidermidis* at concentrations lower than AF alone. Although acetylcysteine is known to be effective in destroying bacterial biofilm [37], its conjugation with AF resulted in lower antibiofilm activity against *S. aureus* and *S. epidermidis* than AF alone. The inclusion of acetylcysteine in AF could potentially interfere with the antimicrobial activity of the pharmacophore against *S. epidermidis* [6]. Moreover, the scarce activity of AF-AcCys against biofilms might be due to the high hydrophobicity of the bacteria after adaptation to biofilm mode of life [38]. In this context, the higher hydrophilicity in comparison to AF alone might reduce the antimicrobial activity of the pharmacophore.

In the study of the potential of NSAIDs on biofilm control [39], it was demonstrated that naproxen sodium was the only NSAID that did not exhibit any antibacterial activity against *E. coli* and *S. aureus* in the range of concentrations up to 2000 μg/mL. On the other hand, the surface of *S. aureus* is more hydrophilic than that of *S. epidermidis*, and this might be attributed to the different chemical composition of the microbial cell walls [40]. Overall, the use of naproxen as ligand imparts a higher lipophilicity compared with the AF-AcCys derivative; this, in turn, might imply a higher interaction leading to a higher antibacterial effect. Additionally, the anti-biofilm activity of AF and its analogues could be related to the inhibition of specific enzymatic pathways. Indeed, several studies have documented the TrxR inhibition activity of AF and/or its analogues. The computed bond strengths excellently corroborate the experimental potency data by showing that the AF-naproxen complex, in which the [AuPEt_3_]^+^ fragment is more easily released or transferred, inhibited biofilms more effectively. Indeed, we envision that a more favourable release of the [AuPEt_3_]^+^ cation from the naproxen complex is prompted by the weaker O-Au(I) bond (hard ligand—soft metal) in this species compared to the stronger S-Au(I) bond (soft ligand—soft metal) in both AF and AF-AcCys. On the other hand, such a hypothesis implies that the investigated metallodrugs penetrate the bacterial cell and bind at specific intracellular targets, thus making the interpretation of the microbiological effects of their administration particularly challenging. The specific chemical composition of the biofilm, as well as the specific structure of the bacterial cell wall, may affect the capability of AF and/or its analogues to reach the cellular compartment in which the release or transfer of the [AuPEt_3_]^+^ fragment must take place.

## 4. Materials and Methods

### 4.1. Reagents

All solvents and reagents were purchased from Sigma-Aldrich (Darmstadt, Germany) and used without further purification. Auranofin was purchased from Cayman Chemical Company. The synthesis of the gold complexes was carried out under nitrogen atmosphere using standard Schlenk techniques. Compounds obtained were stored at −20 °C in the dark. ^1^H, ^13^C{^1^H}, and ^31^P{^1^H} NMR spectra were recorded on a Bruker Avance II 400 spectrometer (^1^H 400.0 MHz, ^13^C 100.6 MHz, and ^31^P 162.0 MHz) with chemical shifts (δ, ppm) reported relative to the solvent peaks of the deuterated solvent. The spectra were processed using the MestreNova software (v12). Elemental analysis (C, H, and N) was accomplished with the VarioMICRO elemental analyser. HR-ESI mass spectra were recorded using an Orbitrap high-resolution mass spectrometer (Thermo, San Jose, CA, USA), equipped with HESI source. Complexes were solubilized in LC-grade anhydrous acetonitrile, just before the analysis. UV–Vis spectra were recorded at room temperature on a Agilent Cary 60 Uv–Vis spectrophotometer using quartz cuvettes (path length: 1 cm).

### 4.2. Synthesis of Compounds

**AF-Napx.** In a Schlenk flask, 100 mg of Et_3_PAuCl (0.29 mmol) was dissolved in 3 mL of EtOH. Then, 98 mg of Ag[NapxCO_2_] (0.29 mmol), synthesized as reported in the literature [23], was added, and the solution was magnetically stirred for 1 h at room temperature in the dark. The reaction mixture was then filtered thorough celite and the solvent removed under reduced pressure. The resulting pale-yellow oil was triturated with Et_2_O for 2 h until a white powder was obtained. The solid was then dried under vacuum and collected. Yield: 87 mg, 55%. CHN: C_20_H_28_AuO_3_P·0.5H_2_O; required: C = 43.41; H = 5.28; N = 0.00. Found: C = 43.18; H = 5.38; N = 0.00. ^1^H-NMR (400 MHz; CDCl_3_): 7.76 (s, 1H; H ar), 7.71 (m, 2H, H ar), 7.56 (dd, *J* = 8.4, 1.7 Hz, 1H, H ar), 7.11 (m, 2H, H ar), 3.92 (s, 3H, CH_3_-O), 3.86 (q, *J* = 7.1 Hz, 1H, CH), 1.80 (dq, *J*_HP_ =18.3 Hz, *J*_HH_ = 7.6 Hz, 6H, CH_2_-P), 1.60 (d, *J* = 7.1 Hz, 3H, CH_3_), 1.17 (dt, *J*_HP_ = 18.8, *J*_HH_ 7.6 Hz, 8H, CH_3_ phosp.). ^31^P{^1^H}-NMR (162 MHz; CDCl_3_): 25.28. ^13^C{^1^H}-NMR (101 MHz; CDCl_3_): 180.18 (COO), 157.29 (C ar), 138.86 (C ar), 133.52 (C ar), 129.46 (C ar), 129.25 (C ar), 127.16 (C ar), 126.86 (C ar), 125.84 (C ar), 118.48 (C ar), 105.71(C ar), 55.40 (CH_3_-O), 48.67 (CH), 19.82 (CH_3_), 17.86 (d; *J*_CP_ = 38.3 Hz; CH_2_-P), 9.08 (CH_3_ phosp.), 9.07 (CH_3_ phosp.). HR-ESI-MS m/z [M−Na]^+^ = 567.13293 (theoretical: 567.13538); [M−K]^+^ = 583.10681 (theoretical: 583.10732).

**AF-AcCys.** This compound was synthesized and characterized following an already- reported procedure [25].

Yield: 97 mg (88%). CHN: C_11_H_25_AuClNNaO_4_PS: required C, 23.86; H, 4.55; N, 2.53. Found: C, 23.86; H, 4.05; N, 2.33. ^1^H-NMR (400 MHz; DMSO-d_6_): 12.43 (s; br; 1H; −COOH); 7.93 (d; 1H; *J*_HH_ = 7.8 Hz; −NH); 4.26 (m; 1H; −CH); 3.11 (dd; 1H; *J*_HH_ = 12.8, 5.2 Hz; −CH_2_); 2,97 (dd; 1H; *J*_HH_ = 12.7, 7.4 Hz; −CH_2_); 1.87 (m; 9H; −CH_3_ Ac. Cyst; −CH_2_ phosphine); 1.13 (m; 9H; −CH_3_ phosphine). ^13^C{^1^H}-NMR (100 MHz; DMSO-d_6_) = 172.94 (COOH); 169.56 (−CO); 57.66 (C-NH); 29.57 (CH_2_-S); 22.99 (CH_3_); 17.46 (d; *J* = 31.4 Hz; −CH_2_ phosphine); 9.38 (−CH_3_ phosphine). ^31^P{^1^H}-NMR (160 MHz; DMSO-d_6_) = 46.71 ([Au(PEt_3_)_2_]^+^); 36.67 (AF-AcCys). HR-ESI-MS m/z [M-H]^−^ = 476.07291 (theoretical: 476.07292).

### 4.3. Measurement of Water–Octanol Partition Coefficient (LogP)

Partition coefficient was determined by the shake-flask method and UV–Vis measurements, according to a previously described procedure [26,41]. All the operations were carried out at room temperature. A stock solution of AF-Napx was prepared in water-saturated octanol. The wavelength corresponding to a maximum of absorption (273 nm) was used for UV–Vis quantitation. The procedure was performed in triplicate (from the same stock solution); LogP value is given as mean ± standard deviation.

### 4.4. Stability Studies in DMSO and Aqueous Solution

The solution stability of AF-AcCyst and AF-Napx was assessed using UV–Vis absorption spectroscopy at room temperature over a 48 h period. Both AF-AcCyst and AF-Napx were evaluated in a mixture of 60% DMSO—40% deionized water at a concentration of 2.23 × 10^−^^4^ M and 2.08 × 10^−^^4^ M, respectively. Spectral absorption data were acquired in the 200–800 nm wavelength range at 10 min intervals for the first hour, followed by 30 min intervals for the duration of the experiment. The degradation percentage was assessed by comparing the absorbance values measured at 264 nm for AF-Napx and 250 nm for AF-AcCys at initial (0 h) and final (48 h) time points. The stability of AF-Napx was also evaluated by ^31^P{^1^H}–NMR experiments recorded at increasing time intervals. Specifically, a known amount of AF-Napx (5 mg, 0.009 mmol) was dissolved in a 6:4 DMSO/D_2_O mixture, and NMR spectra were collected immediately, after 2 h, 4 h, 24 h, and 48 h of incubation at 37 °C. The stability of AF-AcCys has already been reported by ^31^P{^1^H}–NMR experiments in a solution of DMSO/D_2_O [6].

### 4.5. Computational Details

All the calculations were carried out by the Gaussian 16 quantum chemistry package [42]. DFT permits the accurate analysis of reactions of transition metal complexes, in particular, including Au [43,44]. The hybrid range-corrected functional ωB97X-D [45] was used for all optimizations together with the basis set def2SVP basis set [46]. The employed functional is recognized to produce the trustworthy geometries and accurate evaluation of the electronic and solvation energies [47,48]. The frequency computations substantiated the true character of the stationary points and permitted the assessment of zero-point energy and thermal corrections. The employed IEFPCM continuum solvent method produces significantly smaller errors than other continuum models for aqueous free energies of solvation for cations, anions, and neutrals [49], being especially robust for the computation of solution properties requiring precise assessment of solution free energies [50]. The strength of Au-P, Au-S, and Au-O bonds in the studied complexes was characterized via the computation of the corresponding snapping energies, i.e., the energies for the dissociation into unrelaxed fragments (FEs), BDEs, and BDFEs. Fragmentation energies were obtained by subtracting the single-point energies of the two bonding fragments at the geometry taken by the complex from the electronic energy of the whole optimized complex. BDEs/BDFEs are the differences between the enthalpy/free energy of the fully optimized complexes and the enthalpies/free energies of the bonding fragments after relaxation.

### 4.6. Cell Cytotoxicity Assessment: XTT Reduction Assay on A549 Cell Line

Human lung carcinoma epithelial-like A549 cell line (ATCC-CCL 185) (LGC Standards, Sesto San Giovanni, Italy) was cultured in tissue culture flasks in Dulbecco’s modified Eagle’s medium (DMEM) (Euroclone, Pero, Milan, Italy) supplemented with 2 mM L-glutamine, 100 U/mL penicillin, 100 µg/mL streptomycin, and 10% heat-inactivated Fetal Bovine Serum (Euroclone). Cell cultures were maintained in a 5% CO_2_ atmosphere at 37 °C, once grown to confluence cells were harvested using 1× Trypsin-EDTA solution (Euroclone) and split in new flasks.

In vitro cytotoxicity of different compounds AF, AF-Napx, and AF-AcCyst at different concentrations (1, 2, 4, 8, and 16 µM) was evaluated using the XTT reduction assay [51]. AF, AF-AcCys, and AF-Napx were resuspended in DMSO and then diluted in 1× PBS. This assay is based on the reduction of sodium 3′-[1-phenyl-aminocarbonyl)-3,4-tetrazolium]bis(4-methoxy-6-nitro)benzenesulphonic acid (XTT) into formazan by the activity of mitochondrial dehydrogenases. Formazan formation is measured spectrophotometrically, and the product colour intensity can be correlated to the number of viable cells. *N*-methylphenazonium methyl sulphate (PMS), an electron coupling agent, is added to improve the reduction in XTT.

Cells cultured as previously described were resuspended in complete DMEM, then 200 µL of the cell suspension was seeded to a concentration of 1 × 10^4^ cells/well in flat-bottom 96-well microtiter plates and incubated for 24 h at 37 °C in a 5% CO_2_ atmosphere. The next day, culture media were removed and replaced with 100 µL of fresh complete DMEM containing the appropriate concentrations of the compounds to be tested (1, 2, 4, 8, and 16 µM). Culture media alone and 1% (*v*/*v*) Triton X-100 (Sigma-Aldrich, Milan, Italy) were also included as negative and positive controls, respectively. After 24 h of incubation at 37 °C in a 5% CO_2_ atmosphere, 50 µL of freshly prepared XTT/PMS (Glentham Life Sciences, Planegg, Germany) solution was added and the plate was incubated for 3 h at 37 °C in a 5% CO_2_ atmosphere protected from light. Finally, 100 µL of the solution was transferred into a new plate and absorbance at 450 nm was read using a microplate reader (Multiskan^TM^ GO Thermo Scientific, Milan, Italy). Three independent experiments with three replicates each were performed.

Cytotoxicity was presented as the percentage of cell viability with respect to the negative control, and calculated as follows:

%cell viability = (OD 450s)/(OD 450n) × 100

where OD 450s is the absorbance at 450 nm of formazan salt production by A549 cells upon exposure to the test sample.

OD 450n is the absorbance at 450 nm of formazan salt production by A549 cells in the negative control.

The lower the viability value the higher the cytotoxic potential of the compound or material.

Data were expressed as mean ± SD. Statistical analysis was performed using the one-way analysis of variance (ANOVA) followed by the Dunnett’s multiple comparison test, with a significance level of *p* ≤ 0.05 using the GraphPad Instat software (version 6.05 for Windows, La Jolla, CA, USA).

### 4.7. Staphylococcal Strains

Six staphylococcal clinical isolates, previously described [6], were included in the study. In particular, three *S. aureus* and three *S. epidermidis* drug-resistant strains (resistance profile formerly reported [6]) were tested. *S. aureus* MRSA ATCC 43,300 was used as a reference strain. The tested isolates were stored as frozen stocks in cryovials (Greiner Bio-One, Kremsmünster, Austria) at −80 °C. For bacterial cultivation, 250 µL of bacteria from the stock was inoculated in 5 mL of Müller–Hinton broth (MHB) (Sigma-Aldrich, St. Louis, MO, USA) and incubated overnight at 37 °C under agitation. Bacteria were plated on Brain Heart Infusion (BHI) agar (Sigma-Aldrich, St. Louis, MO, USA) for colony counting.

### 4.8. Antimicrobial Susceptibility Testing of Planktonic Bacteria

AF, AF-AcCys, and AF-Napx were tested for their antibacterial activity against the staphylococcal isolates by using broth microdilution assay, according the Clinical and Laboratory Standards Institute (CLSI) guidelines. Briefly, 100 µL of a 10^6^ CFU/mL bacterial suspension were seeded on a 96-well plate round bottom, then 2-fold serial dilutions ranging from 4 to 0.12 µM of AF, AF-AcCys, and AF-Napx previously resuspended in DMSO and diluted in 1× PBS were added, and the cells incubated overnight at 37 °C. A growth control, containing bacteria at a concentration of 10^6^ CFU/mL without the antimicrobial agents, was included. Following the incubation, the minimum inhibitory concentration (MIC), defined as the lowest concentration of antimicrobial agent that inhibits visible growth of a microorganism compared to the untreated control [52], was determined.

### 4.9. Evaluation of Antibiofilm Activity of AF, AF-AcCys, and AF-Napx

The antibiofilm activity of the gold-based compounds was assessed against *S. aureus* and *S. epidermidis* in sessile form. Biofilm was formed on porous glass beads (ROBUVR, Hattert, Germany), following a previously established protocol [53,54]. Porous glass beads were incubated with overnight bacterial culture diluted up to 10^7^ CFU/mL in MHB for 24 h at 37 °C. The ratio between beads and bacterial culture was 1 bead:1 mL. After the formation of the biofilm, the bead samples were washed three times with 1× PBS and then incubated in MHB containing two-fold serial dilutions of AF, AF-AcCys, and AF-Napx (from 8 to 0.5 µM) for 24 h at 37 °C. An untreated control comprising 24 h old biofilm beads incubated in 1 mL of fresh MHB was also included. After incubation, the beads were washed again three times with 1× PBS. The bead samples were transferred into a 2 mL Eppendorf tube containing 1 mL of 1× PBS, then vortexed for 30 s, sonicated for 1 min using a sonication bath, and vortexed again for 30 s. After the mechanical disaggregation of the biofilm-forming cells, the sonication suspension was 10-fold serially diluted in 1× PBS and plated onto BHI agar. Plates were incubated for 24 h at 37 °C for colony counting to determine the bacterial concentration expressed as CFU/mL. Each test condition was evaluated with 3 independent biological replicates.

The minimum biofilm bactericidal concentration (MBBC) and the minimum biofilm eradication concentration (MBEC) of each antimicrobial agent were determined for the examined strains. The MBBC is defined as the lowest concentration of antibiotic required to kill the 99.9 (i.e., 3 log10 CFU/mL) of the bacteria in a biofilm condition compared with growth control [55], while the MBEC is defined as the lowest concentration of antimicrobial required to eradicate the sessile cells (0 CFU/mL on plate) [56].

### 4.10. Investigation by Scanning Electron Microscopy of S. aureus and S. epidermidis in Sessile Form

The effect of the antimicrobial agents at sub-MBBC on the structure of the biofilm of *S. aureus* ATCC43300 and *S. epidermidis* 7 was investigated by scanning electron microscopy (SEM). For *S. aureus* ATCC43300, biofilm was formed on glass slides by incubating each slide with 400 µL of overnight bacterial culture diluted up to 10^7^ CFU/mL in MHB for 24 h at 37 °C. After incubation, the samples were washed three times with 1× PBS and then incubated in MHB containing different concentrations of AF (2 µM), AF-AcCys (2 µM), and AF-Napx (2 µM) for 24 h. For *S. epidermidis*, after the biofilm formation on porous glass beads as previously described, the samples were treated with AF (1 µM), AF-AcCys (2 µM) and AF-Napx (0.25 µM) for 24 h at 37 °C. Once the incubation period was finished, both substrates glass slides and glass beads were washed 3 times with 1× PBS and then fixed and dehydrated, as previously described by Ferretti and colleagues [54]. Briefly, 2.5% glutaraldehyde solution diluted in 0.1 M sodium cacodylate buffer (Sigma-Aldrich, St. Louis, MO, USA), was added to fix each sample for 1 h at room temperature. Samples were then washed 3 times for 2–3 min in 0.1 M sodium cacodylate buffer. Next, biofilms were gradually dehydrated by incubating the samples at different concentrations of ethanol (one time with 30%, 50%, 70%, 85%, and 95%, and two times with 100%) for 10 min. Dehydrated biofilm samples were then exposed for 4 min to a solution composed of concentrated hexamethyldisilazane (HMDS) (Sigma-Aldrich, St. Louis, MO, USA) mixed with 100% ethanol (1:1 *v*/*v*). Finally, concentrated HMDS was added for a further of 4 min to the samples. After overnight drying on filter paper, the samples containing the formed biofilm were coated by sputtering with platinum to make them electrically conductive. A field emission scanning electron microscope (FEI Company Quanta 450 FEG, Hillsboro, OR, USA) was employed to acquire the micrographs.

## 5. Conclusions

In conclusion, AF might be used for treating biofilm-associated bacterial infections. Moreover, AF conjugated with naproxen might be applied for *S. epidermidis*-embedded biofilm, particularly in the treatment of device-associated infections where both infection and inflammation are present.

## Figures and Tables

**Table 1:** MIC values of AF, AF-AcCys, and AF-Napx for *S. aureus* and *S. epidermidis* isolates.

Strains		MIC (µM)	
	AF	AF-AcCys	AF-Napx
*S. aureus* ATCC43300	2	0.5	2
*S. aureus* 10	0.5 *	0.5 *	0.25
*S. aureus* 11	0.25 *	0.25 *	0.25
*S. aureus* 12	0.5 *	0.5 *	2
*S. epidermidis* 2	≤0.12 *	2 *	0.25
*S. epidermidis* 6	≤0.12 *	2 *	0.25
*S. epidermidis* 7	≤0.12 *	>2 *	0.5

* Previously published [6].

**Table 2:** MBBC of AF, AF-AcCys, and AF-Napx for biofilm of *S. aureus* and *S. epidermidis*.

Strains	AF (µM)		AF-AcCys (µM)		AF-Npax (µM)	
	MBBC	MBEC	MBBC	MBEC	MBBC	MBEC
*S. aureus* ATCC43300	8	8	8	>8	8	>8
*S. aureus* 10	4	4	8	>8	8	>8
*S. aureus* 11	0.5	2	>8	>8	>8	>8
*S. aureus* 12	4	>8	4	8	8	>8
*S. epidermidis* 2	2–4	2–4	>8	>8	1	1
*S. epidermidis* 6	4	8	>8	>8	1	2
*S. epidermidis* 7	4	4	8	>8	1	1

**Table 3:** Metal ligand bond strengths in complexes: fragmentation energy (FE), bond dissociation energy (BDE), and bond dissociation free energy (BDFE). In kcal/mol.

Complex	Bond	FE	BDE	BDFE
AF	Au-P Au-S	61.5 54.1	56.2 50.9	46.0 40.8
AF-AcCys	Au-P Au-S	62.3 59.4	57.5 56.4	45.1 44.1
AF-Napx	Au-P Au-O	73.3 42.0	68.9 38.4	56.8 27.4

## Data Availability

The datasets generated for this study are not publicly available due to privacy restrictions, but they are available on request to the corresponding author.

## Supplementary Materials

The following supporting information can be downloaded at: https://www.mdpi.com/article/10.3390/antibiotics14020118/s1, Figure S1: ^1^H-NMR (400 MHz; CDCl_3_) spectrum of AF-Napx; Figure S2: ^31^P-NMR (160MHz; CDCl_3_) spectrum of AF-Napx; Figure S3: ^13^C-NMR (101MHz; CDCl_3_) spectrum of AF-Napx; Figure S4: Collection of UV-Vis spectra of AF-Napx in DMSO-H_2_O (60–40%) over the course of 48 h; Figure S5: Collection of UV-Vis spectra of AF-AcCys in DMSO-H_2_O (60–40%) over the course of 48 h. Figure S6: HR-ESI mass spectrum of AF-Napx; Figure S7: HR-ESI mass spectrum of AF-AcCys.
