# Supplementary material for: Antimicrobial and Antibiofilm Activity of Auranofin and Its Two Derivatives Bearing Naproxen and Acetylcysteine as Ligands Against Staphylococci
Source: Antibiotics (Basel). 2025 Jan 23;14(2):118. doi: 10.3390/antibiotics14020118 (PMC11851661; doi:10.3390/antibiotics14020118)
Supplement: Supplementary file 1 [file antibiotics-14-00118-s001.zip › antibiotics-3411461-supplementary.pdf]

Supporting Information

Antimicrobial and antibiofilm activity against staphylococci of auranofin and its two derivatives bearing naproxen and acetylcysteine as ligands

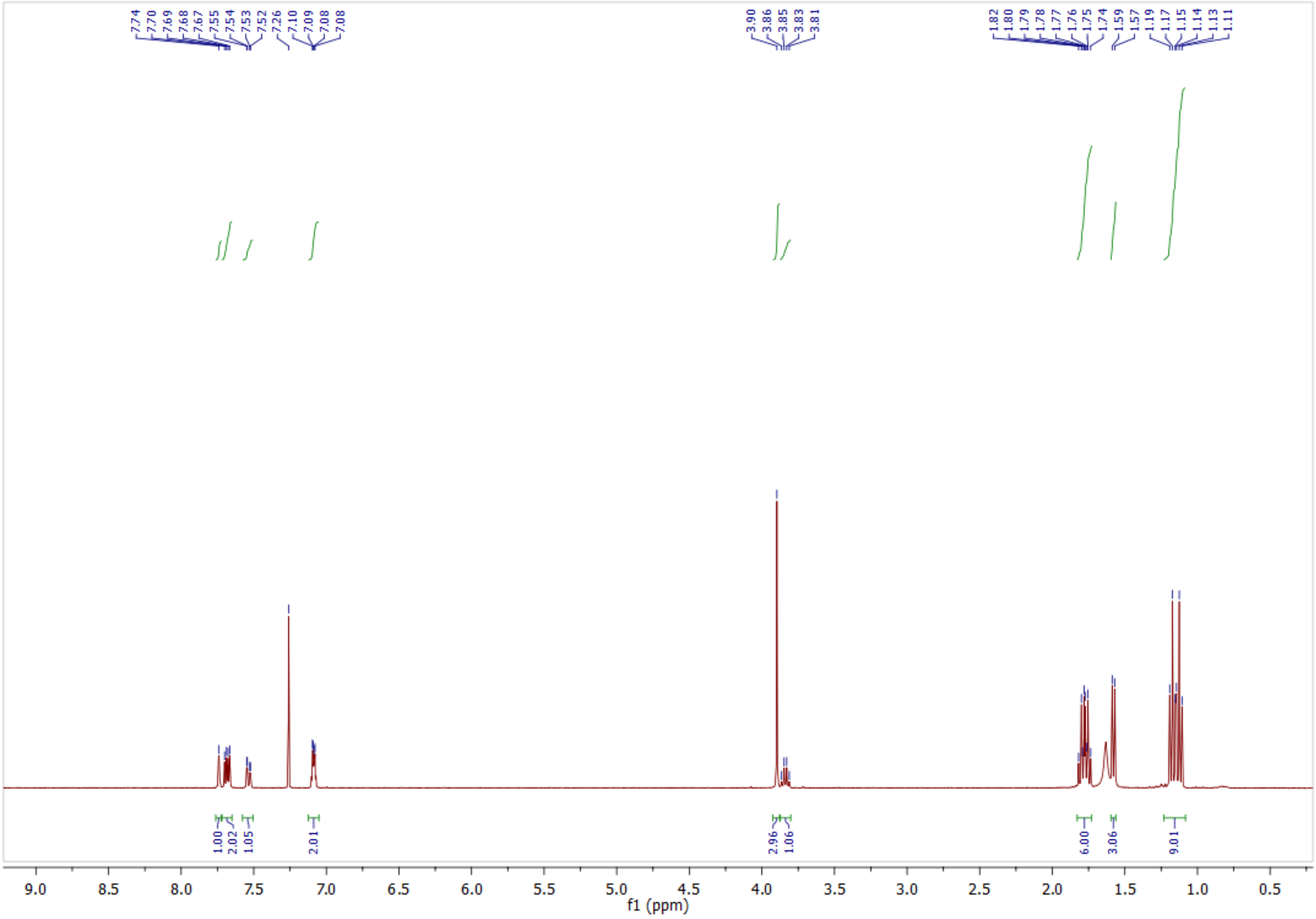

*Figure S1 ^{1}H-NMR (400MHz; $CDCl_3$) spectrum of **AF-Napx**.*

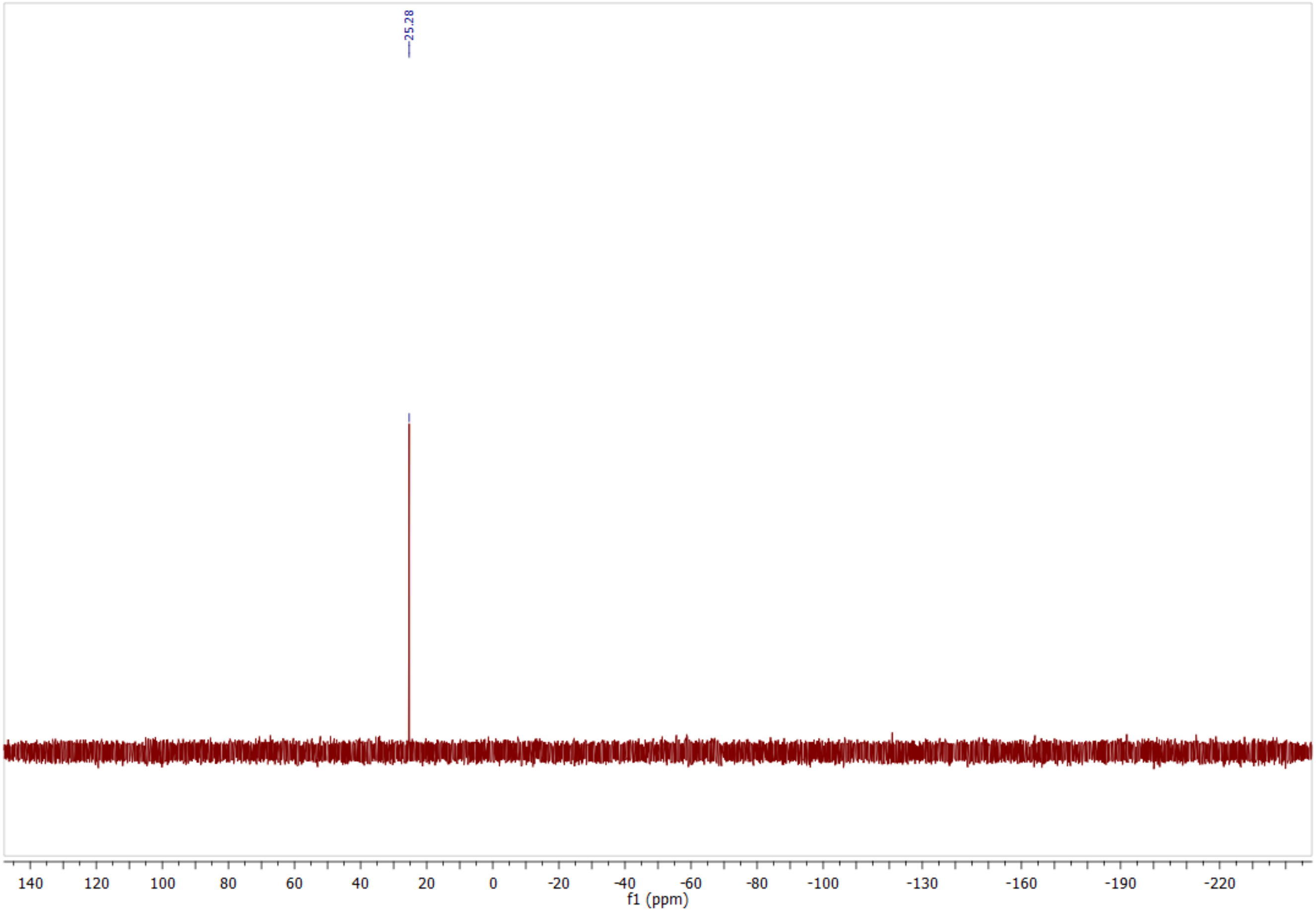

*Figure S2 ^{31}P-NMR (160MHz; $CDCl_3$) spectrum of **AF-Napx**.*

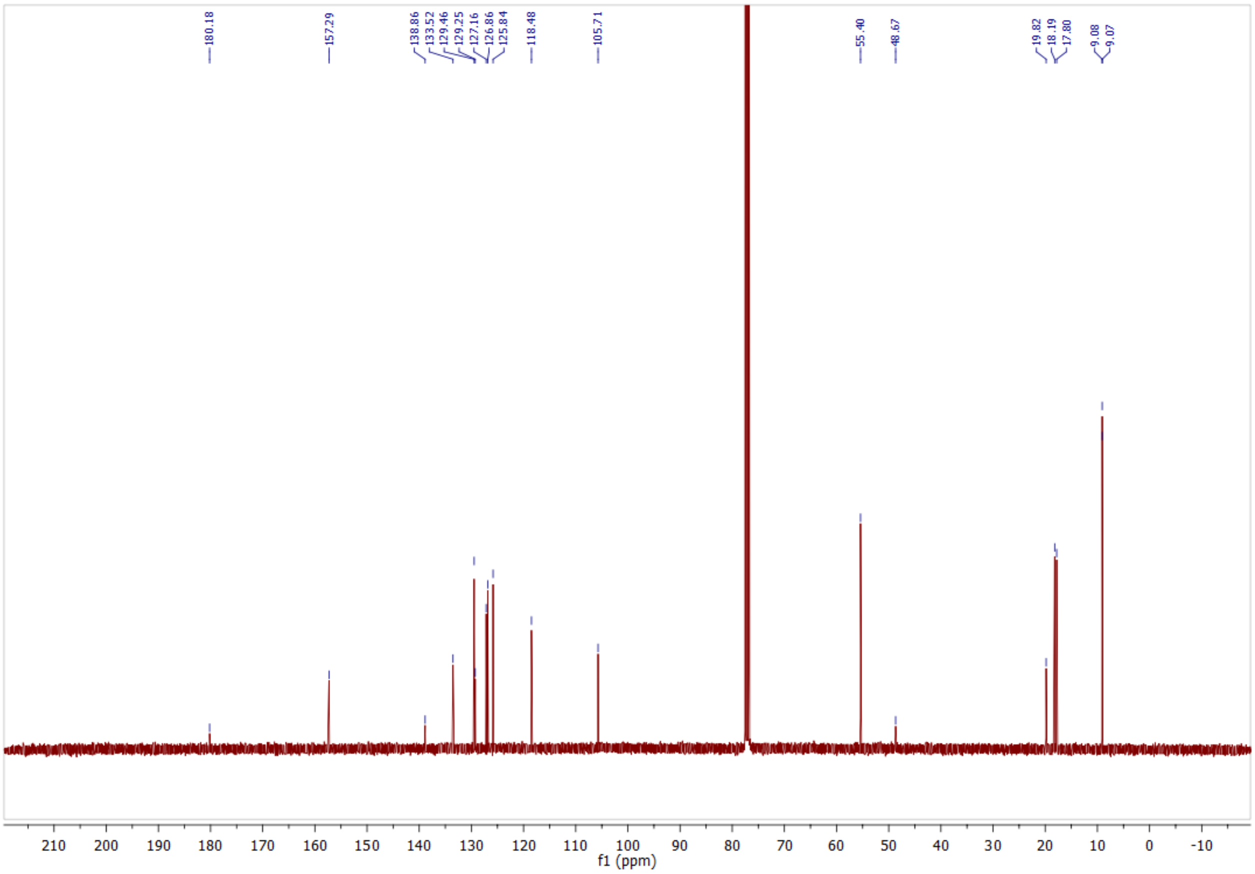

*Figure S3 ^{13}C-NMR (101MHz; $CDCl_3$) spectrum of **AF-Napx**.*

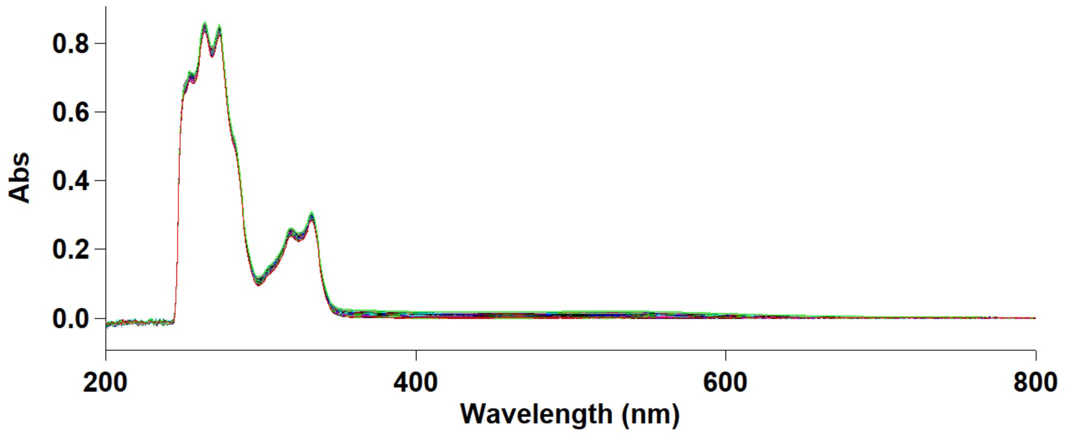

Figure S4 Collection of UV-Vis spectra of AF-Napx in DMSO-H_2O (60 – 40 %) over the course of 48 h.

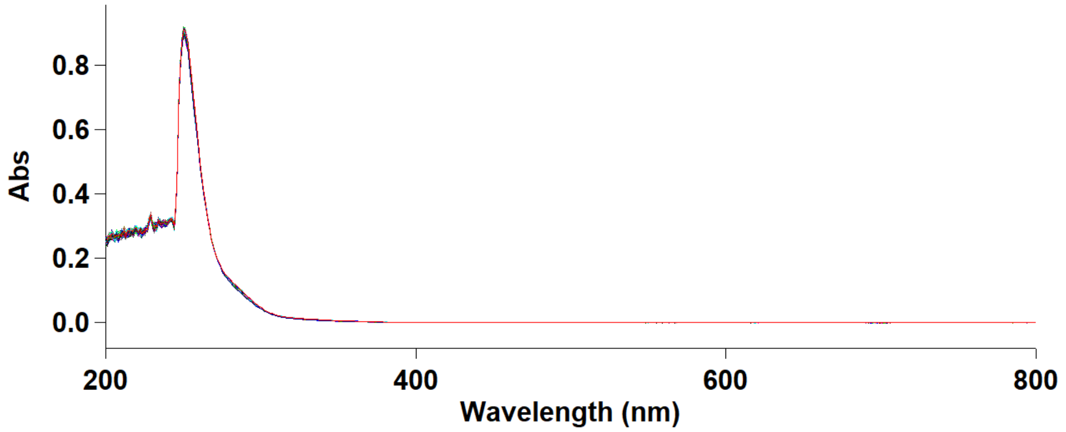

Figure S5 Collection of UV-Vis spectra of AF-AcCys in DMSO-H_2O (60 – 40 %) over the course of 48 h.

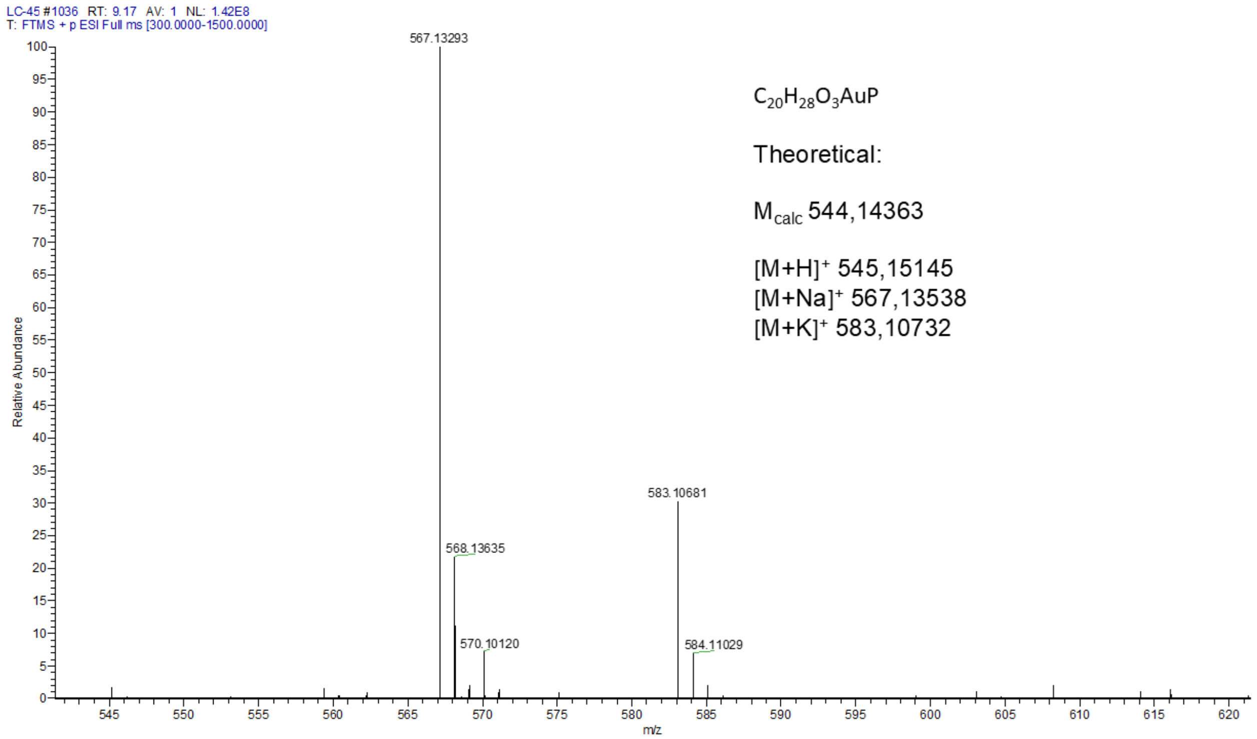

Figure S6 HR-ESI mass spectrum of AF-Napx.

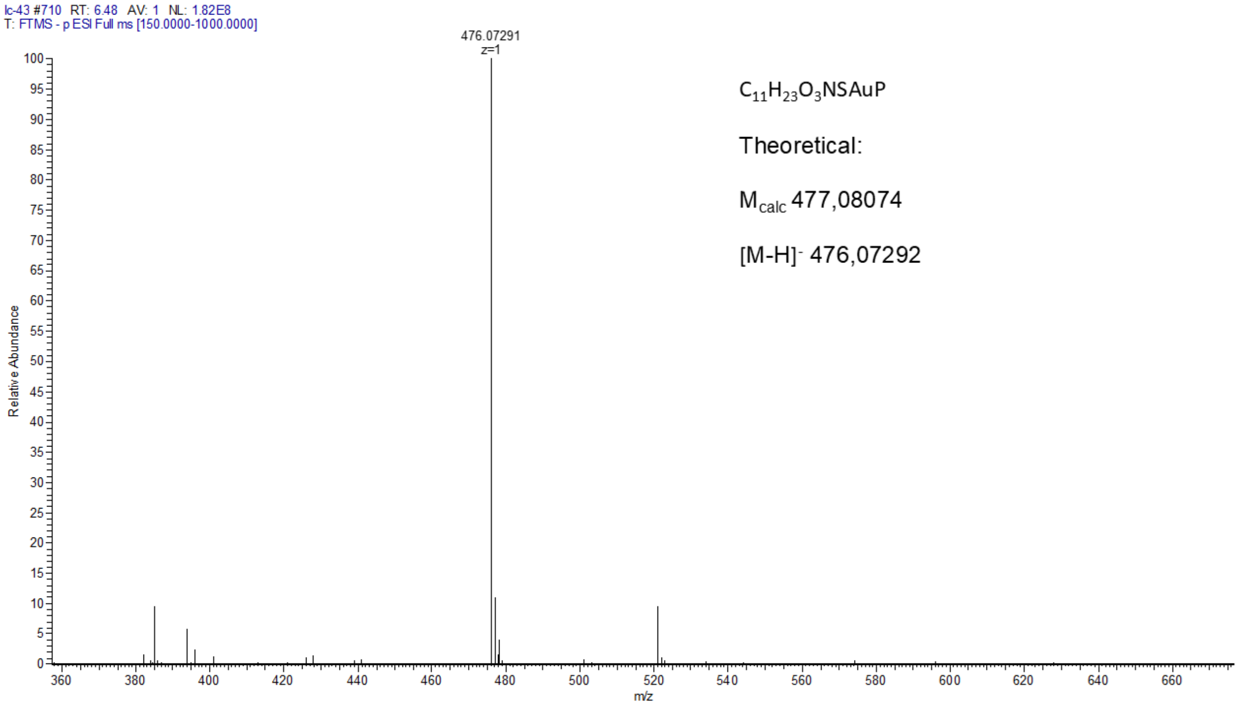

Figure S7 HR-ESI mass spectrum of AF-AcCys.
